# Total knee arthroplasty improves the quality-adjusted life years in patients who exceeded their estimated life expectancy

**DOI:** 10.1007/s00264-020-04917-y

**Published:** 2021-01-15

**Authors:** Michele Palazzuolo, Alexander Antoniadis, Jaad Mahlouly, Julien Wegrzyn

**Affiliations:** 1 Department of Orthopedic Surgery, Lausanne University Hospital – CHUV, Lausanne, Switzerland; 2 Hôpital Orthopédique, Centre Hospitalier Universitaire Vaudois – CHUV, Avenue Pierre Decker, 4, CH-1011 Lausanne, Switzerland

**Keywords:** Total knee arthroplasty, Elderly patients, Quality-adjusted life years, Complication, Mortality

## Abstract

**Purpose:**

Total knee arthroplasty (TKA) is the treatment of choice for end-stage osteoarthritis though its risk-benefit ratio in elderly patients remains debated. This study aimed to evaluate the functional outcome, rates of complication and mortality, and quality-adjusted life years (QALY) in patients who exceeded their estimated life expectancy.

**Methods:**

Ninety-seven TKA implanted in 86 patients who exceeded their estimated life expectancy at the time of TKA were prospectively included in our institutional joint registry and retrospectively analyzed. At latest follow-up, the functional outcome with the Knee Society Score (KSS), rates of complication and mortality, and QALY with utility value of EuroQol-5D score were evaluated.

**Results:**

At a mean follow-up of three ± one years, the pre- to post-operative KSS improved significantly (*p* < 0.01). The rates of surgical and major medical complications related to TKA were 3% and 10%, respectively. The re-operation rate with readmission was 3% while no TKA was revised. The 30-day and one year mortality was 1% and 3%, respectively. The pre- to one year post-operative QALY improved significantly (*p* < 0.01). The cumulative QALY five years after TKA was four years. Assuming that these patients did not undergo TKA, their cumulative QALY at five years would have been only two years.

**Conclusion:**

TKA is an effective procedure for the treatment of end-stage osteoarthritis in patients who exceeded their estimated life expectancy. TKA provided significant improvement in function and quality of life without adversely affecting overall morbidity and mortality. Therefore, TKA should not be contra-indicated in elderly patients based on their advanced age alone.

## Introduction

End-stage knee osteoarthritis (OA) is a major cause of disability and functional decline with loss of independence in elderly patients [1]. Total knee arthroplasty (TKA) is an effective surgical treatment that provides relief of pain, functional restoration, and improvement in overall quality of life in patients suffering from knee OA [2, 3]. However, as any major surgical procedure, TKA could be challenging when performed in patients who exceeded their estimated life expectancy regarding potential occurrence of post-operative medical- or surgical-related complications that may affect the overall morbidity and mortality [4, 5]. Previous studies reported that total joint arthroplasties in elderly patients were associated with higher risk of post-operative adverse events with longer in-hospital stay duration and higher readmission rate [6–9]. In addition, using a risk-estimation model, Klausing et al. [10] demonstrated that every year of life increases the probability of post-operative adverse events after TKA by 2%. However, demographic projections demonstrated that individuals older than 85 years represent the most rapidly growing cohort of patients in the USA that will account for 2.3% of the population in 2030 and 4.3% in 2050 [11]. Therefore, the demand for TKA from patients who exceeded their estimated life expectancy is projected to grow accordingly confronting total knee surgeons to new paradigm and challenge [12, 13].

Previous studies reported significant improvement in QALY after total hip and knee arthroplasty in global cohorts of patients [14–17]. However, to our knowledge, no study was dedicated to evaluate the improvement in quality of life that could be expected after TKA in patients who exceeded their estimated life expectancy at the time of surgery in order to balance the risk-benefit ratio of this elective procedure in these specific patients [18, 19]. Therefore, this retrospective study on prospectively collected data aimed to evaluate the functional outcome, rates of complication and mortality, and QALY in a consecutive cohort of patients who exceeded their estimated life expectancy at the time of TKA.

## Patients and methods

### Patients

From 2015 to 2018, patients who underwent TKA for end-stage primary knee OA were prospectively included in our institutional joint registry. Patient’s informed consent and Institutional Review Board approval were obtained before initiating this study (CER-VD# 2020-00832). Through review of the clinical and operative reports, a continuous series of 97 TKA performed in 86 patients who exceeded their estimated life expectancy at the time of surgery (i.e., ≥ 83 years in 2015 in Switzerland [20]) was then identified and retrospectively analyzed at latest follow-up.

**Fig. 1:**
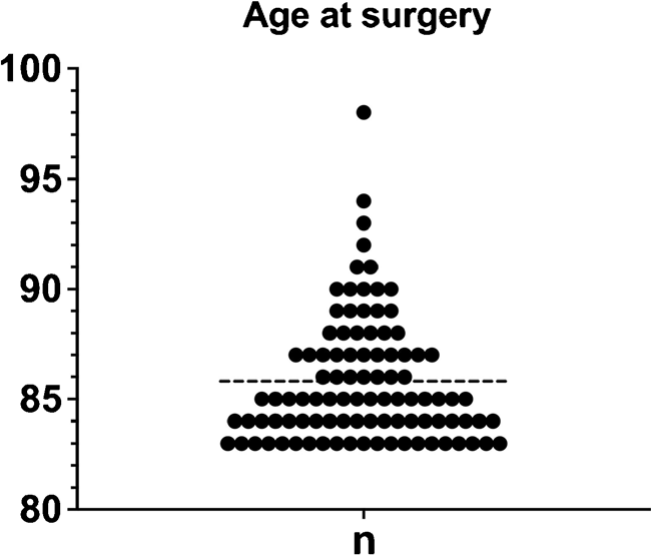
Patients’ age at surgery: patient number by age and mean of age at surgery

There were 62 women and 24 men. The mean body mass index was 27 ± 4 kg/m^2^. The mean age at the time of surgery was 86 ± three years (Fig. 1). Particularly, 11 patients (13%, two men, nine women, 11 TKA) were older than 90 with the oldest patient being a 98-year-old woman. According to the American Society of Anesthesiologists (ASA) physical status classification, 46 patients (53%) were ASA II and 40 patients (47%) were ASA III. No patient was ASA IV or presented with terminal illness at the time of surgery. The main patients’ comorbidities are reported in Table 1. Before TKA, all of the patients were active, independent in their daily living activities, and cognitively preserved. In addition, none of them was living in a skilled nursing facility pre-operatively. The mean follow-up of the entire series was of three ± one years at the latest evaluation or until death.

**Table 1:** Main comorbidities of the patients before TKA

Medical comorbidities	Number of patients (%)
Hypertension	56 (65)
Diabetes	17 (20)
Chronic anemia	16 (19)
Supraventricular arrhythmia	15 (17)
Chronic renal failure	15 (17)
Ischemic heart disease	13 (15)
Thromboembolic disease	10 (12)
Obesity	10 (12)
Chronic obstructive pulmonary disease	7 (8)
Cerebrovascular accident	4 (5)
Obstructive sleep apnea	3 (4)

### Peri-operative care and surgical procedure

Before surgery, all the patients were evaluated for chronic anaemia according to recommendations of our institutional blood management program and optimized when haemoglobin was < 10 mg/L with the objective to obtain a pre-surgical haemoglobin level of 13 mg/L [21]. Twenty-one patients (24%) received intravenous iron supplementation and/or blood transfusion before TKA. In patients suffering from diabetes, blood glucose was also evaluated and then optimized when required with short-acting subcutaneous insulin regimen from 48 hours before surgery to 48 hours post-operatively.

All the TKA were performed by or under the direct supervision of a senior fellowship-trained arthroplasty surgeon through a conventional medial parapatellar approach without tourniquet in supine position under spinal (67 TKA, 69%) or general (30 TKA, 31%) anaesthesia. A conventional antibiotic prophylaxis with cefuroxime (or clindamycin in case of allergy) was intravenously administered before skin incision. Intravenous tranexamic acid was systematically administered intra-operatively. A conventional primary cemented postero-stabilized total knee system with patellar resurfacing was used and implanted with the use a conventional technique with mechanical alignment of the knee. No TKA required additional procedure such as hardware removal, combined osteotomy, or bone reconstruction. Before knee closure, an antalgic peri-articular infiltration with ropivacaine and a haemostatic intra-articular injection of tranexamic acid were systematically performed. Then, the surgical incision was closed without drainage and the wound was covered with a sterile hydrocolloid dressing for seven days.

A conventional rapid rehabilitation protocol that includes multimodal pain management, immediate full weight–bearing ambulation with a walker or crutches, and complete range of motion knee mobilization was systematically prescribed and adapted to the patient as tolerated. Postoperative blood management was performed according to the guidelines of the British Committee for standards in blood transfusion for acute anaemia [22]. The transfusion threshold of haemoglobin was 10 mg/L in patients with cardiovascular diseases and 7 mg/L in the other patients, and adapted according to symptoms of acute anemia. Oral laxative medication was systematically administered to prevent constipation related to bed resting and opioids. A bladder catheter was used only as needed in patients with acute post-operative urinary retention. All the patients received a thrombosis prevention with lower limb compression stocking and thromboprophylaxis with subcutaneous enoxaparin during 30 days. Patients were discharged out of the hospital when pain was adequately controlled with oral medication and independent ambulation achieved with two crutches and knee flexion ≥ 90°. Skin staples were removed at the 15th post-operative day at our outpatient clinic facility.

### Evaluation

Operative and anesthesiology reports were reviewed for assessment of the operative time from the skin incision to wound dressing, intra-operative bleeding by measuring fluid accumulation in the suction device after subtracting irrigation and weighing gauze swabs, occurrence of intra-operative complication, and the need for post-operative intermediate (IMCU) or intensive (ICU) care unit. After TKA, the duration of in-hospital stays and discharge disposition (i.e., patient’s home, acute rehabilitation facility, or in-hospital death) were reported. Medical reports were reviewed for post-operative in-hospital medical and surgical complications.

Patients returned for post-operative follow-up visits at three months, six months, one year, and annually thereafter. Patients underwent a physical examination by the operating surgeon. Plain long leg, Merchant view, antero-posterior, and lateral radiographs of the affected knee were obtained. The functional outcome was evaluated with the Knee Society Score (KSS; part 1—Knee score, part 2—Function score and Total KSS). The minimal clinically important difference (MCID) was 9 for the KSS knee score and 10 for the KSS function score [23]. Patient’s pre-operative and post-operative health status was measured with the EuroQol-5D (EQ-5D) score [24]. Each question of the EQ-5D was scored and then converted to EQ-5D utility score [14]. An EQ-5D utility score of 1 equates to one QALY. One QALY equates to one year in perfect health. QALY point values range from 1 (perfect health) to 0 (death). The cumulative postoperative QALY at an endpoint was calculated as:

$$ ``\mathrm{cumulative}\ \mathrm{QALY}=\mathrm{years}\ \mathrm{of}\ \mathrm{life}\ \mathrm{after}\ \mathrm{TKA}\times \mathrm{patient}\ \mathrm{survivorship}\times \mathrm{postoperative}\ \mathrm{EQ}-5\mathrm{D}\ \mathrm{utility}\ \mathrm{score}" $$

The time point value of QALY at 1 year after TKA equals the EQ-5D utility score at one year and allows to quantify the pre- to one year post-operative improvement in QALY after TKA [15, 25]. Finally, the post-operative complication, patient readmission, TKA re-operation or revision, and mortality data were collected through retrospective chart review.

### Statistical analysis

Data are presented as mean ± standard deviation. Comparison of the pre- to post-operative continuous and quantitative variables was performed using two-sample *t* tests with a level of evidence set at *p* < 0.05. Patient survivorship analysis was performed using Kaplan-Meier curves with 95% confidence intervals (CI) using death at the endpoint. Statistical analyses were performed using the SPSS version 22 software (SPSS Inc., Chicago, IL).

## Results

### Peri-operative outcomes

The mean intra-operative blood loss was 450 ± 200 mL. The mean operative time was 118 ± 24 minutes. Intra-operative medical complications related to anesthesiology occurred during 11 TKA (11%), being arrhythmia in 6 TKA (6%) and hypotension in 5 TKA (5%). Intra-operative surgical complications occurred during two TKA (2%), being two medial condylar fractures that were treated with internal fixation using lag screws without compromising the TKA procedure. IMCU was required after 12 TKA (12%) due to pre-existing medical comorbidities in patients with ASA III status. ICU was required after one TKA (1%) to monitor a supraventricular arrhythmia.

At patient’s discharge, the disposition was a transfer to an acute rehabilitation facility after 79 TKA (81%) and patient’s home after 17 TKA (18%). The mean in-hospital stay duration was nine ± four days and was significantly longer in patients transferred to an acute rehabilitation facility compared to those discharged at home (9 ± 4 vs. 6 ± 3 days, respectively; *p* < 0.01).

### Complications and mortality

Post-operative medical complications related to anesthesiology included urinary retention after 12 TKA (12%) conducted under spinal anaesthesia and acute confusion after six TKA (6%) conducted under general anaesthesia. Minor medical complications related to the surgical procedure were post-operative anaemia requiring blood transfusion after 13 TKA (13%). A major life-threatening medical complication was reported after ten TKA (10%) (Table 2).

**Table 2:** Major medical complications that occurred after TKA

Major medical complication	Number of TKA (%)
Acute renal failure	4 (4)
Pneumonia	1 (1)
Pulmonary embolism	1 (1)
Stroke	1 (1)
Myocardial infarction	1 (1)
Congestive heart failure	1 (1)
Upper gastrointestinal hemorrhage	1 (1)
Total	10 (10)

After discharge, surgical complications were reported after three TKA (3%) which required patient readmission and TKA re-operation. There were one wound dehiscence that was managed with debridement, one severe haematoma that was managed with drainage, and one periprosthetic distal femoral fracture without implant loosening related to a fall that was managed with open reduction and internal fixation. Importantly, no TKA was revised at latest follow-up.

**Fig. 2:**
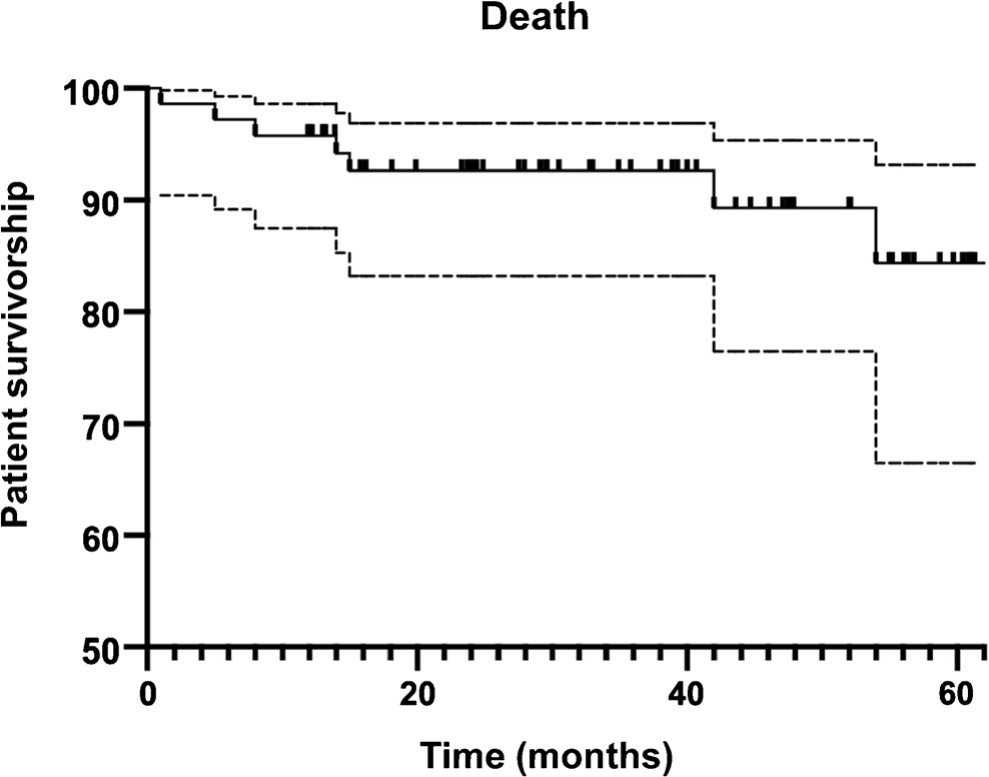
Kaplan-Meier curve showing survivorship of elderly patients who exceeded their estimated life expectancy at the time of TKA

Regarding mortality, one case of in-hospital death (1%) was reported in a 94-year-old male patient with ASA III status who deceased of congestive heart failure and acute renal failure nine days after TKA. No other patient died within the first post-operative month. Therefore, the 30-day mortality was 1%. One after TKA, three patients died from causes that were not related to TKA. Therefore, the one year mortality was 3%. The patient survivorship at one year and five years after TKA was 96% (95% CI: 84–99%) and 84% (95% CI: 67–93%), respectively (Fig. 2).

### Functional outcome and QALY

At a mean follow-up of three ± one years, the pre- to post-operative total, knee, and function KSS scores improved significantly (*p* < 0.01) (Table 3). Importantly, no patient experienced worst function or pain at latest follow-up after TKA.

**Table 3:** Pre- to post-operative comparisons of the Knee Society Score (KSS)

KSS	Pre-operative (mean ± SD)	Post-operative (mean ± SD)	*p*
Part 1—Knee	48 ± 16	87 ± 11*	< 0.001
Part 2—Function	50 ± 16	91 ± 14**	< 0.001
Total KSS	99 ± 26	178 ± 22	< 0.001

*Minimal clinically important difference = 9 [23]. **Minimal clinically important difference = 10 [23]

The pre- to one year post-operative QALY improved significantly from 0.58 ± 0.25 to 0.90 ± 0.11 (*p* < 0.01). Importantly, this improvement in QALY was significant for all the patients. No patient obtained worst EQ-5D utility score one year after TKA. The cumulative QALY five years after TKA was four years. Assuming that these elderly patients with end-stage knee OA did not undergo TKA with no change in their pre-operative value of EQ-5D utility score over time, their cumulative QALY at five years would have been only two years.

## Discussion

End-stage knee OA is a leading cause of disabling pain, functional impairment, and decline in quality of life [1]. Along with the worldwide population aging and increase in life expectancy, the number of elderly patients undergoing TKA and their functional demand in daily living activities are growing [11, 12]. Due to pre-existing comorbidities and sometimes-low physiological reserves, the risks of post-operative adverse event and 30-day mortality were reported to be significantly higher after total hip and knee arthroplasty when performed in elderly patients [5–10]. However, the benefit of TKA in terms of improvement in quality of life in these patients is sparsely evaluated in literature, mainly through global cohorts of patients [15, 16, 26]. Indeed, no previous study to our knowledge was specifically dedicated to evaluate the improvement in quality of life after TKA in patients who exceeded their expected life expectancy at the time of surgery. This lack of knowledge confronts the surgeon with a difficult shared decision-making process when balancing the risk-benefit ratio of TKA in these particular patients. Therefore, this study was dedicated to evaluate the functional outcome, rates of complication and mortality, and QALY in a consecutive cohort of knee OA patients who exceeded their estimated life expectancy (i.e., ≥ 83 years in 2015 in Switzerland [20]) at the time of TKA. The most important finding of this study was that the cumulative QALY five years after TKA was four years. Indeed, the pre- to post-operative improvement in functional outcome was significant for all the patients, with no patient experiencing worst function or pain at latest follow-up. Importantly, assuming that these patients did not undergo TKA, their cumulative QALY at five years would have been only two years. Therefore, the benefit of TKA was to offer to these patients the equivalent of two more years of life in perfect health with respect to their pre-operative knee OA condition. In addition, the pre- to one year post-operative improvement in QALY was 0.32 that was higher than the improvement previously reported in other studies evaluating TKA in global cohort of patients, ranging from 0.17 to 0.27 [15, 26]. Therefore, this difference highlights the fact that any improvement in functional independence and pain relief provided by TKA in elderly patients has stronger positive effects on their overall quality of life when compared to global cohorts that included young patients. This conclusion is even more important to be emphasized with the knowledge that the remaining life expectancy of 83-year-old individuals in the global population in Switzerland is projected to be seven years according to the Federal Statistical Office [27].

Nevertheless, the main concern with elective TKA in elderly patients remains the increased risks of complication and mortality related to pre-existing medical comorbidities and limited physiological reserves. In our study, the readmission rate was 3%. All the readmissions were related to non-specific surgical complication that occurred after three TKA (3%) leading to re-operation. Importantly, the surgical complications were not related to surgical site infection or implant mechanical failure with no TKA being revised at latest follow-up. In addition, no readmission for medical complication was observed. These findings contrast with previous studies reporting 30-day readmission rates up to 7% related to post-operative medical complications or infection of the surgical site [8, 9, 28, 29]. Pre-operatively, all the patients were evaluated for chronic anaemia and optimized when required with intravenous iron supplementation and/or blood transfusion with the objective to obtain a pre-surgical haemoglobin level of 13 mg/L [21]. Although chronic anaemia is a common comorbidity in elderly patients, Klausing et al. [10] demonstrated that pre-operative haemoglobin level was a significant and independent predictor of the overall risks of post-operative complication, admission in ICMU/ICU, and mortality after TKA. In addition, Noticewala et al. [30] demonstrated that every ten year augmentation in patient age increased twice the probability of blood transfusion leading to a 30% rate of blood transfusion in patients over 80 years. In our study, we found that blood transfusion and admission in ICMU/ICU were both required in 13% of the TKA. This lower blood transfusion rate reported in our study could be explained by a systematic pre-operative patient’s blood management program that aimed to identify and correct pre-operative anaemia. This could also explain our rates of 13% for admission to IMCU or ICU and 1% for 30-day mortality that were lower than those of previous similar series reporting rates up to 17% and 3%, respectively [10, 14, 31–34]. In our study, the mean in-hospital stay duration was nine days and was significantly longer in patients transferred to an acute rehabilitation facility compared to those discharged at home. This was in accordance with previous studies reporting in-hospital stay duration ranging from eight to 11 days after TKA in elderly patients [35–37]. However, the difference of three days we observed between these two groups of patients was mainly related to the delay to obtain a bed in a geriatric rehabilitation facility. Importantly, no patient required a transient or definitive skilled nursing facility after TKA. This finding contrasts with other studies that reported a transfer rate to skilled nursing facility as high as 14% [8, 38, 39]. This could indicate that the functional independence improved after TKA and that this procedure did not affect the overall morbidity of the patients evaluated in our cohort.

This study presented with some limitations. First, this study was based on patient demographics and life expectancy projections in Switzerland. Therefore, a generalization of our conclusions to the elderly patients from other countries might not be applicable. Second, TKA were performed in elderly patients who were active, functionally independent for daily living activities, and cognitively preserved. Therefore, our results might not correspond to those of patients who do not meet the criteria mentioned above. Third, this study was retrospective in design and included all the patients with end-stage knee OA that were selected and referred to our clinic from corresponding primary care physicians or rheumatologists. Therefore, this study might have not included some elderly patients that were not referred to our clinic due to a poor health condition, despite severe knee OA.

## Conclusion

TKA is an effective procedure for the treatment of end-stage knee OA in patients who exceeded their estimated life expectancy. TKA provided significant improvement in function and quality of life without adversely affecting the overall morbidity and mortality. Particularly, this study showed that the benefit of TKA was to provide to these patients the equivalent of two more years of life in perfect health with respect to their pre-operative knee OA condition. Therefore, TKA should not be contra-indicated in elderly patients based on their advanced age alone, especially for those who are active, functionally independent, and cognitively preserved. However, elderly patients with multiple pre-existing comorbidities should be carefully evaluated and optimized in the pre-operative period with a TKA performed in an institution with appropriate support regarding the potential need for IMCU or ICU.

## Data Availability

All the data and material are saved in an anonymized repository file folder and available upon request.
